# Da art of storytellin': hip-hop epistemology, hip-hop theorization, and the discursive continuity of black cultural expression in/through sport

**DOI:** 10.3389/fspor.2026.1944513

**Published:** 2026-09-17

**Authors:** Sayvon J. L. Foster, Max Klein

**Affiliations:** 1 Department of Health, Sport, and Exercise Sciences, University of Kansas, Lawrence, KS, United States; 2 School of Business, Linfield University, McMinnville, OR, United States

**Keywords:** black intellectual traditions, black studies, counterstorytelling, critical cultural studies, cultural communication, diaspora, meaning-making, sociocultural constructivism

## Abstract

Black cultural expression (BCE) is inextricably linked to sport through Black athletes, coaches, consumers, and practitioners – resulting in Blackness being infused through the sporting complex, e.g., operations, participation, branding, and more. While scholarship exploring Blackness within sport has contributed greatly to the shared understanding of Black representation, resistance, uplift, and a myriad of other topics – the framing is either through a historical review or timely (episodic) analysis. While both are valuable, minimal scholarship has explored BCE through the lens of discursive continuity or the persistence of messaging across time, space, and context. As a result, a valuable element of BCE is left understudied, while simultaneously reasserting that Blackness is understood through time-bound lenses. To address this gap, this Perspective introduces Hip-Hop Epistemology (HHE) and Hip-Hop Theorization (HHT) as complementary frameworks for exploring and understanding BCE in/through sport, specifically discursive continuity. Following the foundations set by Jazz Theory, Blues Theory, and other Black-centric communicative frameworks, HHE and HHT position hip-hop as a form of Black discourse, social critique, messaging, and sense-making. In turn, aligning hip-hop with Blackness in/through sport allows for the tracing of shared language, messaging, and experience despite differences in time, space, and industry context. Emphasizing the discursive continuity between hip-hop and BCE in/through sport illustrates the connectedness of the Black experience to the sporting complex.

## Introduction

“You are now about to witness the strength of street knowledge” - Dr. Dre on S*traight Outta Compton* by N.W.A.

The connection between sport, the Black community, and music is a long-standing tradition that has shaped generations – from James Weldon Johnson penning *Lift Every Voice and Sing*; to Paul Robeson embodying the intersection of sport, scholarship, and artistic performance; to the intertwined nature of contemporary Black sporting culture and hip-hop. Focusing on the intersection of hip-hop and Black culture, Rose (1) argues that this musical genre serves as a tool to amplify Black voices and actively push back against hegemonic systems and social structures. Even further, the language, aesthetics, and underlying logics of the subgenres (political hip-hop, golden era, horrorcore, and more), provide insights into the broader Black experience across time, space, and context. Similar frameworks, such as Blues Commentary, Jazz Studies, and Trap Critical Theory, have outlined how Black musical (and cultural) expression operates as a site of analysis, interrogation, and interpretation (2–5). However, despite these contributions, hip-hop is still relatively understudied and analyzed within sport scholarship. Addressing this gap offers sport scholarship a culturally-situated lens to better explore and understand how Black athletes, fans, and practitioners make meaning their sport experiences through the use of cultural forms like language, aesthetics, and cultural expression. Doing so illuminates the discursive continuity of Black cultural expression by drawing attention to shared messaging, logics, and experiences across distinct spaces, reflecting a consistency in the Black experience.

As Wilson (6) details in his interpretation of Du Bois, a discursive theory of racial identity asserts that these identities are socially constructed through language and discourse. More specifically, discursive continuity, as it relates to BCE, is the persistence of messaging, logics, and narratives despite changes in time, space, or communicator/audience (7, 8). Through the use of cultural artifacts, specifically forms of media, shared discourse and understanding can be used to create counterarchives that center communal ways of knowing and shape responses to dominant narratives (8). With attention to hip-hop and sport, an emphasis on discursive continuity offers an identity-centric approach towards understanding how language, aesthetics, and cultural logics are rearticulated across time, space, and social context. To note, Foster (9) aligned GZA's (member of the Wu-Tang Clan) critique of hegemonic Whiteness within the music industry to commentary provided in Black athlete-led media (BALM) platforms. Despite over three decades between these cultural artifacts, the shared messaging illustrates how hegemonic Whiteness remains embedded across industry, but also how Black cultural creators leverage their art and space as forms of resistance. Through connecting these cultural spaces, further opportunities are developed to understand and trace how BCE exists and persists despite temporal and spatial boundaries.

Given the focus on language and communication, qualitative methods underpinned with identity-centric frameworks are ideal for this focus on continued discourse between sport, hip-hop, and BCE, such as thematic analysis (10) critical cultural analysis (11), and critical discourse analysis (12). Units of analysis can range from lyrical content, interviews/ discourse, aesthetic artifacts (e.g., music videos and album covers), and more. Notably, analysis of discursive continuity within the intersection of hip-hop and sport center BCE and the Black experience. For example, researchers can explore shared messaging/experiences between Big L's “The Enemy” and the discursive landscape surrounding the transfer portal, NIL (Name, Image, and Likeness), and increased player platforming. Through this analysis, researchers could highlight the persistence of anti-Black carceral logics communicated through lyrical content and aligned with discourse provided by both coaches/administrators and Black athletes. In turn, this analysis could highlight how the anti-Black carceral logics imposed upon Big L by the New York Police Department (NYPD) are replicated by high-powered coaches/administrators on Black college athletes. The similarity in discourse exposes the larger issue of both anti-Black carcerality and inability to accept shifting power and resource dynamics. As a result of its potential in critical applications, discursive continuity research can also aid in answering Shaw and Hoeber's (13) call for novel qualitative research in sport management. Considering the relationship between sport and hip-hop through the concept of discursive continuity moves analysis beyond time-bound, episodic moments to deeper examinations of how Black-centered understandings of power, hegemony, and the social world persist and transform across contexts. Foster (9) illustrated this by aligning GZA's commentary on hegemonic Whiteness in the music industry with BALM commentary regarding the same force within the sport media ecosystem. From a broader perspective, the proposed Big L study offers a valuable pathway to explore (a) the persistence of anti-Black carceral logics and (b) the understanding and response to these logics by Black athletes. The emphasis on sport and hip-hop highlights how shared identification, understanding, and responses exist across spatial and temporal boundaries.

Ultimately, the purpose of this perspective paper is to formally introduce Hip-Hop Epistemology (HHE) and Hip-Hop Theorization (HHT) as valuable frameworks for better understanding and exploring the discursive continuity of BCE in/through sport through hip-hop. By expanding the scope of hip-hop as a cultural art form, aesthetic elements, and reflections of sociocultural and sociopolitical understanding, HHE and HHT are essential to understanding the shared discursive landscape despite differences in time, location, and context.

## Understanding and expanding black cultural expression in sport

While BCE and hip-hop is grounded in the Black American perspective the connection to the broader Black Diaspora must be acknowledged. Leaning into the Legacies and Dimensions of African Culture (LDAC) established by Boutte et al. (14), BCE reflects shared cultural elements like communalism, expressive individualism, oral tradition, movement, and more. The existence of these elements across the diaspora posits that while grounded in a Black American lens, a broader application to BCE is applicable (15). Similarly, while hip-hop is seen as a Black American musical form, the localized developments of the genre – including Gullah rap, Caribbean rap, and British Drill – reflects the diasporic nature of BCE. Not only is a discursive continuity present within hip-hop, the underlying ethos of the genre speaks to a shared understanding of BCE across geographic, historical, and social contexts.

Singer (16) noted the presence of epistemological racism within the study of sport that leaves considerable gaps in the inclusion of race and race-centric frameworks. Since then, a range of scholars have contributed to a growing catalog of sport scholarship centered on Blackness and BCE. Broadly, Hylton (17) used critical race theory (CRT) to outline how race is manifested in and through the sporting complex. Similarly, Keaton and Macaulay (18) noted the institutionalization processes that embed race and racialization within sport (e.g., Blackness, Whiteness, and anti-Blackness). With attention to sport media, scholars have noted how the sport media complex promotes idealized stereotypes of Black athletes by aligning their athletic output to perceived urban authenticity or ghettocentrism (19, 20). From a consumer perspective, McGreggor (21) and Foster (22) explored how the Black sport consumption is shaped by shared cultural underpinnings of communalism, pride, and uplift. Collectively, the intentional inclusion of Blackness and BCE demonstrates the value and necessity of race-centric approaches as sources of knowledge, meaning-making, and broadstroke theoretical development.

Given these contributions, the field of sport is positioned to continue expanding the bounds of Blackness and BCE in sport. Through following the foundations set by Jazz Studies, Blues Commentary, and Trap Critical Theory, the inclusion and development of a hip-hop driven framework offers a timely opportunity to expand how BCE is understood. With attention to discursive continuity, or the persistence of messaging, this framework expands the scope and utility of hip-hop as a broader reflection of the Black experience.

## Why hip-hop? black cultural expression as discourse

Hip-hop is uniquely positioned to serve as a framework for understanding discursive continuity because it operates as more than a musical genre. Throughout the history of hip-hop, this musical style has operated as a communicative space that allows for the documentation of the Black experience, critique of social institutions, establish identity, and create alternative realities (1). Understanding this point, hip-hop should be positioned beyond the sonic and commercialized elements, but as a broader collection of language, aesthetics, imagery, symbols, and cultural logics. Given the totality of hip-hop as a cultural artifact, the messaging and impact extends far beyond the sound systems and circulates across multiple domains like education, health, politics, and sport. The ability for hip-hop to find meaning and alignment across spaces is reflective of discursive continuity and further affirms the consistencies seen within the Black experience.

This consistency is not accidental but reflective of the broader Black discursive tradition. Existing frameworks have been developed to outline relationships between Black cultural forms and lived experience (2–5), it is important to continue that tradition with hip-hop. Understanding hip-hop as social discourse allows for isolated moments and topics to be explored as elements of a broader string of experiences and perspectives – similar to sport. At the core, both spaces operate as social institutions, resulting in a collection of experiences, values, and norms that shape and are shaped by Black life. Tracing the discourse provides a legibility to both sport and hip-hop, while also offering critical insights into how messaging exists throughout the Black discursive tradition.

## Hip-hop epistemology (HHE)

Hip-Hop Epistemology (HHE) has firm roots within education and pedagogical studies, often through the justification of art and discursive forms as valid sites of meaning-making and communicative understanding. Dolberry (23) offered a foundational introduction into HHE where they emphasized the inseparability of hip-hop culture and ways of knowing from the communities who are foundationally shaped by this cultural form. Building upon this inseparability, Ashe (24) offered a critical and necessary development where the underpinnings of hip-hop were actively integrated into the understanding of HHE, e.g., Black-centered narrative, social critique, counterstorytelling, reclamation of voice, and the deconstruction of White-normative standards of communication/understanding. In detail, Ashe (24) presented HHE as:

- liberatory mental orientation that positions a person to create, validate, critique, and share knowledge and information through Hip-Hop's aesthetics, elements, and sociopolitical perspectives. Possessing a Hip-Hop epistemology offers a person the ability to decode the systems and behaviors that are window dressed as ideals, deconstructing those “ideals” to reveal the basic elements that put Black folks at a disadvantage: White-dominant ideology, master narratives, and racist systems. Hip-Hop epistemologies prepare educators to analyze and disrupt these elements in real time with Hip-Hop traditions, signaling to others what they are encountering, and then delivering a counter story that assesses, critiques, and displaces incomplete narratives about racial groups that have been historically marginalized. (p. 8–9)

Collectively, HHE expands the understanding of hip-hop beyond music. Consideration for the aesthetics, linguistics, history, and normative cultures of hip-hop introduces a vibrant landscape of knowledge, meaning, and discovery. In turn, this epistemological approach works to center Blackness, Black ways of knowing, and the expansive Black discursive landscape. Through this framework, critical and liberatory scholarship can be designed to actively name, center, and amplify recurring patterns of discourse and meaning-making across sport, media, music, and everyday life.

## Hip-hop theorization (HHT)

Hip-hop theorization stems from Rose's (1) argument that hip-hop shares an explicit connection to the social world through the utilization of language, narrative, and the Black American experience. In turn, hip-hop serves as a discursive cultural art form that actively engages in social analysis, cultural (re)production, and critiques of power. In detail, Rose (1) posits that engaging in the spectrum of hip-hop results in complex and varied discourse surrounding racism, exploitation of state power, existence/resistance, and social mobility. As a result, hip-hop exists and should be understood as a theory-building cultural art form.

Building upon Rose's (1) early development of hip-hop theorization, Sachs (25) offered a timely intervention regarding the utilization of hip-hop across scholarship. Specifically, Sachs argued against an oversimplified and surface-level inclusion of hip-hop into scholarly inquiry - reflecting Pilon's (26) call for a depth ontology that considers the visibility and invisibility of causal social dynamics. Within HHT, this depth ontology requires an understanding that the spectrum of hip-hop (dress, speech, history, and more) provides a complex narrative extending beyond a surface level exploration of lyrics and imagery. Sachs (25) argued that a fully-developed HHT “recognizes the complexity of intersecting social, political, economic, cultural, historical, and ideological forces that work to continually (re)articulate hip-hop” (p. 7).

Collectively, HHT positions hip-hop as a legitimate and valuable site for the theorization of the social, cultural, and political dynamics that shape Black life. Rather than reducing hip-hop to a collection of lyrics and symbolic markers, HHT emphasizes the discursive insights provided through this art form. This deep-level theorization offers scholars the toolkit to explore how this cultural art form generates and communicates meaning across interconnected sights of BCE.

## Toward a hip-hop informed understanding of discursive continuity in sport

The concept of discursive continuity is relatively understudied across sport scholarship, especially when focusing on the Black sporting experience. Discursive continuity reflects the persistence of messaging, lived experience, and the logics that shape Black life. Aligning Black discourse in/through sport with a cultural art form like hip-hop expands the scope of understanding and provides a deeper and clearer legibility for the Black experience and condition. HHE and HHT offer a framework for understanding how BCE exists and persists across sport, media, music, and the Black lived experience. The following four (4) propositions provide a guiding framework for those seeking to explore the discursive continuity of BCE in/through sport.

*Proposition 1: Sport and hip-hop are sites of Black knowledge production*. This proposition serves as the foundation for a hip-hop informed understanding and exploration of Black discursive continuity. Both the Black sporting space and the hip-hop landscape have experienced waves of intentional devaluation, minimization, and silencing – resulting in both being positioned devoid of insight and solely framed as performance (25, 27). To note, Smith and Hattery (28) argued that the integration of Black athletes (and Black people, broadly) into the White-dominant sporting landscape challenged ideological stances and embedded Blackness across the sport ecosystem. Similarly, Oates (29) outlined how Jay-Z leaned heavily into the presumed authenticity of urban life, hip-hop, and basketball to push for the New Jersey Nets to relocate to Brooklyn. These examples further support that the totality of hip-hop (language, aesthetics, and logics) are integral towards understanding the full spectrum of Blackness (1, 30).
*Proposition 2: Hip-hop is a shared interpretive language across Black cultural domains.* Drawing from Blues Commentary, Jazz Studies, and the linguistic traditions of Black art, hip-hop also exists as a form of documentation, resistance, counternarrative, and sense-making (3–5). Notably, Northcutt et al. (31) connected the lyrics of imagery of Jay-Z's *The Life of O.J.* to explain the realities of Black life – both in and out of sport. Similarly, Bell and Kidd (32) explored how the use of namedropping (referencing an athlete or celebrity within a rap lyric) positively and negatively contributed to the growth of baseball role models for Black youth. A HHE and HHT foundation builds upon these approaches by positioning hip-hop as an interpretive language, as opposed to being understood through varying lenses. Through this proposition, the scholarly inquiry of discourse within and across hip-hop engages a depth ontology that considers location, history, lived experience, and sociopolitical landscapes - resulting in a comprehensive understanding of “social, political, economic, cultural, historical, and ideological forces that work to continually (re)articulate hip-hop” (25, p. 7).
*Proposition 3: Black cultural expression through hip-hop is undergirded by Black resistance and survival.* Discursive continuity should not be mistaken or reduced to an exact replication of language, but understood as a collection of common themes reworked to account for time, space, and context. Just as jazz and blues reflected elements of resistance, counternarrative, and the realities of the racialized Black experience, so does hip-hop (3, 4). For example, Harrison et al. (33), engaged in a 2Pac analysis where both his lyrical content and social commentary were used to explore how he made sense of the gendered and racialized performances of Black male athletes. Leaning into counternarratives, cultural-embeddedness, and a rejection of White normative standards is essential towards understanding the connection between hip-hop and the sociopolitical understandings of Black people (5).
*Proposition 4: Discursive continuity is both cultural and political.* The existence and persistence of language across time, space, and context is an essential element of discursive continuity. As asserted by Rose (1), hip-hop operates as a mirror to society and creates a holistic discursive landscape offering insights into the social, cultural, political, and material realities of Black life. Similarly, sport operates as a social institution that actively shapes these elements of Black life by shaping and being shaped by social norms, belief systems, and reprimand/reward systems (34). As explained by Harrison and Coakley (35), the relationship between sport and hip-hop reflects a shared landscape of communicated values, expression of identity, and the expansion of cultural artifacts. As such, discursive continuity should not be reduced to just a regurgitation of language, but a deliberate rearticulation of BCE across social, cultural, and political contexts.

## Implications for sport scholarship

Building upon the expansion and legitimization of this discursive landscape, an application of HHE and HHT within sport scholarship dismantles longstanding barriers regarding who is seen and considered as producers of knowledge. Jenkins (36) emphasized the importance of shifting the understanding of genius/knowledge creation through the inclusion of hip-hop and rap, just as Singer (16) urged the discipline to address longstanding trends of epistemological racism that limits whose voices are included and how their racialized self is considered in scholarship. In doing so, discourse from Black voices and communities transitions from simple identification to deep-level tracing. Rather than framing discourse and cultural expression as isolated or connected to broader themes, illuminating the continuity between that discourse and hip-hop draws attention to recurring narratives, culturally-embedded logics, and the interconnected nature of the Black experience. In turn, the discourse provided by both hip-hop and BCE in sport are dually understood as historically situated and reflective of the broader Black experience and condition.

In addition to scholarly inquiry, this HHE and HHT grounding of sport scholarship offers considerable practical implications. By framing the spectrum of sport as inextricably linked to the social world (37), this hip-hop underpinning helps explain how perspectives, aesthetics, belief systems, and utilizations of power (or lack thereof) are integrated into the sporting complex. For example, Sudre et al. (38) explored how Afro-French basketball players replicated presentations and playing styles reminiscent of the hip-hop culture seen within the NBA, much to the chagrin of their coaches. Both the replication from the players and dismay from coaches outline broader social dynamics regarding BCE, acceptability, and rejection. Extending beyond players and coaches, HHE and HHT offers key insights into social media communication and discourse – largely through the concepts of signifyin' and contagion (39, 40). Signifyin', or the display of Blackness, works in tandem with contagion by increasing the reception and reach of BCE across the digital landscape. For consumers, these digital spaces offer the opportunity to share experience and perspective across the boundaries of geography, age, and diasporic cultural divides. In turn, practitioners can leverage this discourse as a way to better understand the interests and perspectives of their consumer base. Broadly, attention to these continuities allows practitioners, consumers, leaders, and participants to navigate sport through a culturally-embedded lens (41).

Collectively, the inclusion of HHE and HHT within sport scholarship deepens the understanding of sport as a social institution - primarily through an alignment in discourse, expression, and culturally-embedded logics. This hip-hop informed understanding of sport and sport discourse encourages a timely development in how Black cultural meaning exists and persists across sport and the broader Black community. In doing so, sport scholarship is better situated to center the complexity, continuity, and dynamic nature of BCE.

## Conclusion

The intersection of sport, hip-hop, and Black cultural expression provides a foundation to use cultural artifacts to expand the understanding of Black life (35). While their 2020 *Sociology of Sport Journal* special issue set the foundation for the inquiry and utilization of hip-hop in sport research, Harrison and Coakley (35) hoped for future scholars to expand the bounds of their contribution. Building upon this invitation, the four propositions developed in this Perspective transform hip-hop from a cultural artifact to a source of theorization and knowledge production by emphasizing discursive continuity between hip-hop and the Black sporting experience. In doing so, the framework offers scholars the ability to trace how Black cultural expression persists across time, space, and context - further amplifying the shared understanding of Black life.

Ultimately, the value of HHE and HHT centers on repositioning sport and hip-hop as linked spaces of Black cultural expression. The history, values, and ways of knowing connected to the Black community undergird both spaces. In turn, a focus on discursive continuity contributes to nuanced understandings of both not as episodic, but continuations of shared interpretations of Black life. Recognizing the continuities allows scholars to shift the understanding and framing of Black cultural expression from momentary to constant rearticulations of Black sensemaking. Through this hip-hop informed approach, HHE and HHT contribute to the theoretical and epistemological toolkit available to scholars seeking to better explore, understand, and explain the Black sporting experience.

## Data Availability

The original contributions presented in the study are included in the article/Supplementary Material, further inquiries can be directed to the corresponding author.
